# Serum Levels of Interleukin 10 (IL-10) in Patients with Type 2 Diabetes

**Published:** 2011-10-01

**Authors:** N Yaghini, M Mahmoodi, Gh R Asadikaram, Gh H Hassanshahi, H Khoramdelazad, M Kazemi Arababadi

**Affiliations:** 1 Department of Biochemistry, Rafsanjan University of Medical Sciences, Rafsanjan, Iran; 2 Molecular-Medicine Research Center, Rafsanjan University of Medical Sciences, Rafsanjan, Iran; 3 Department of Microbiology, Hematology and Immunology, Rafsanjan University of Medical Sciences, Rafsanjan, Iran; 4 Infectious and Tropical Disease Research Center, Rafsanjan University of Medical Sciences, Rafsanjan, Iran

**Keywords:** Interleukin 10, Type 2 diabetes

Dear Editor,

The frequency of diabetes mellitus is increasing and it is expected that this disorder will affect 300 million people in 2025.[1] It has been suggested that diabetes is an immune dependent disease in which the pattern of cytokine expression is changed.[2] As an example, in type 2 diabetes, the monocytes of peripheral blood produce more inflammatory cytokines than those from normal patients.[2] The association of IL-10 in immunological disorders such as multiple sclerosis,[3][4] nephrotic syndrome[5][6] and type-1 diabetes[7][8] is well established. The key role of IL-10 is to work as the main inhibitory cytokine against the action of inflammatory cytokines such as IL-12. Based on evidence suggesting that immune responses may be important in inducing type 2 diabetes,[9] this study was designed to evaluate serum levels of IL-10 in type 2 diabetes. Peripheral blood samples were collected from 131 type 2 diabetic patients and 120 healthy controls. The patient and control groups were matched for sex and age. IL-10 serum level was measured using ELISA kit (eBioscience, Spain) in both groups. The differences in variables were analyzed by student t tests. Results of our study showed that the mean IL-10 serum level was 9.53±2.27 and 16.11±2.27 pg/ml in type 2 diabetic patients and control groups, respectively. Statistical analysis showed that the difference was significant (p<0.005). Our findings indicated a significant difference between IL-10 serum levels in type 2 diabetic patients compared to healthy controls. Other researchers also showed same results such as Eric VE et al., 2002 who indicated that the serum levels of IL-10 decreased in type 2 diabetic patients compared to controls.[10] Based on this fact, it may be concluded that low serum levels of IL-10 can be considered as a risk factor of type 2 diabetes.
